# Splicing Analysis of *MYO5B* Noncanonical Variants in Patients with Low Gamma-Glutamyltransferase Cholestasis

**DOI:** 10.1155/2023/8848362

**Published:** 2023-07-27

**Authors:** Li Wang, Yi-Ling Qiu, Kuerbanjiang Abuduxikuer, Neng-Li Wang, Zhong-Die Li, Ye Cheng, Yi Lu, Xin-Bao Xie, Qing-He Xing, Jian-She Wang

**Affiliations:** ^1^The Center for Pediatric Liver Diseases, Children's Hospital of Fudan University, National Children's Medical Center, Shanghai, China; ^2^Children's Hospital of Fudan University, National Children's Medical Center and Institutes of Biomedical Sciences of Fudan University, Shanghai, China; ^3^Shanghai Key Laboratory of Birth Defect, Shanghai, China

## Abstract

Biallelic *MYO5B* variants have been associated with familial intrahepatic cholestasis (FIC) with low serum gamma-glutamyltransferase (GGT). Intronic or synonymous variants outside of canonical splice sites (hereinafter referred to as noncanonical variants) with uncertain significance were identified in *MYO5B* posing a challenge in clinical interpretation. This study is aimed at assessing the effects of these variants on premessenger RNA (pre-mRNA) splicing to improve recognition of pathogenic spliceogenic variants in *MYO5B* and better characterize the *MYO5B* genetic variation spectrum. Disease-associated *MYO5B* noncanonical variants were collected from the literature or newly identified low GGT cholestasis patients. *In silico* splicing predictions were performed to prioritize potential pathogenic variants. Minigene splicing assays were performed to determine their splicing patterns, with confirmation by blood RNA analysis in one case. Eleven (five novel) noncanonical variants with uncertain significance were identified. Minigene splicing assays revealed that three variants (c.2090+3A>T, c.2414+5G>T, and c.613-11G>A) caused complete aberrations, five variants (c.2349A>G/p.(=), c.4221G>A/p.(=), c.1322+5G>A, c.1669-35A>C, and c.3045+3A>T) caused predominant aberrations, and three variants (c.4852+11A>G, c.455+8T>C, and c.2415-6C>G) had no effect on pre-mRNA splicing. Patient-derived RNA analysis showed consistent results. Based on our results, eight variants were reclassified as likely pathogenic and three as likely benign. Combining the clinical features and the above analysis, the diagnosis of *MYO5B*-associated FIC could be made in three new patients. In conclusion, we characterized the splicing patterns of *MYO5B* noncanonical variants and suggest that RNA analysis should be routinely included in clinical diagnostics to provide essential evidence for the interpretation of variants.

## 1. Introduction

Variation in *MYO5B*, encoding the motor protein myosin Vb (MYO5B), can cause microvillus inclusion disease and/or familial intrahepatic cholestasis (FIC) with normal/low serum gamma-glutamyltransferase (GGT<100 IU/L) activity [1–4]. Microvillus inclusion disease is a rare enteropathy leading to intractable, life-threatening diarrhea in neonates, which is usually associated with null *MYO5B* variants, such as nonsense, frameshift, and canonical splice site [4–7]. FIC, as a recently identified phenotype, is less lethal and seems to be a continuous phenotypical spectrum, including persistent, recurrent, and transient cholestasis [4]. *MYO5B*-associated FIC is usually associated with nonnull variants, and intronic or synonymous variants outside of canonical +1/+2 or −1/−2 splice sites (hereinafter referred to as noncanonical variants) with the potential to affect splicing are occasionally identified [7]. To date, *MYO5B* noncanonical variants account for approximately 10% of the reported disease-associated variants in FIC patients, and approximately 15% of reported *MYO5B*-associated FIC patients carried at least one such variant [7]. Variant of uncertain significance (VUS) reporting is also becoming commonplace for the routine use of next-generation sequencing technologies in clinical practice. The pathogenicity of canonical splicing variant requires little supporting evidence, while establishing the pathogenicity of other noncanonical variants relies on more supportive evidence. Unfortunately, the supportive evidence is not available specifically for these noncanonical variants in *MYO5B*, which poses a challenge in clinical diagnostics.

To address this issue, we collected *MYO5B* noncanonical variants with uncertain significance both from the literature and new patients with suspected *MYO5B*-associated low GGT cholestasis. *In vitro* cell-based specific minigene splicing assays, as well as *in vivo* patient-derived blood RNA splicing, were performed to evaluate their potential damaging effect on splicing. This study is aimed at achieving a more precise classification of noncanonical variants in *MYO5B*, thereby enhancing our understanding of the genetic variation spectrum in *MYO5B* and improving genetic diagnosis.

## 2. Methods

### 2.1. Subjects

Preexisting whole-exome sequencing (WES) data was reanalyzed in low GGT intrahepatic cholestasis patients without a molecular diagnosis who were referred to Children's Hospital of Fudan University from January 2015 to March 2022. Inclusion criteria were patients with biallelic *MYO5B* potential pathogenic variants and at least one of them was noncanonical variant with the potential to affect splicing. *MYO5B* potential pathogenic variants were defined as follows: (1) allele frequency less than 0.001 in the Genome Aggregation Database (gnomAD, https://gnomad.http://broadinstitute.org, last accessed January 30, 2022) and (2) variants, except for nonsense, frameshift, canonical splice sites, and in-frame variants, should be predicted to be deleterious or splice affecting by at least 1 of 5 *in silico* tools (detailed in the following methods). Patients were excluded from this study if they met one of the following criteria: (1) the same genotype was also present in the normal family members and (2) presence of pathogenic or likely pathogenic variants classified following the American College of Medical Genetics and Genomics (ACMG) guidelines [8] and a recently proposed ACMG point system [9] in other low GGT intrahepatic cholestasis-related genes (including at least *ATP8B1*, *ABCB11*, *NR1H4*, *TJP2*, *USP53*, *VPS33B*, *VIPAR*, LSR, *CYP27A1*, *CYP7B1*, *HSD3B7*, and *AKR1D1*). The flowchart of patient selection is shown in Figure S1. A total of 11 *MYO5B* noncanonical variants were studied, including 5 newly identified and 6 previously reported (retrieved using electronic searches on PubMed database; search terms: *MYO5B*, *MYO5B* variants, and cholestasis; last search was undertaken on 31 March 2022). Minigene splicing assays were performed on all selected variants, and 2 canonical splice site variants were used as positive controls for the assay. Blood RNA splicing analysis was also investigated if samples from patients were available.

### 2.2. Nomenclature and *In Silico* Analysis of Variants

All variant nomenclature was checked by the Mutalyzer website (https://www.mutalyzer.nl/) with reference sequence NM_001080467.3 (NP_001073936.1). PROVEAN (http://provean.jcvi.org/index.php), Polyphen2 (http://genetics.bwh.harvard.edu/pph2/), SIFT (https://www.sift.co.uk/), MutationTaster (http://www.mutationtaster.org/), and REVEL (https://sites.google.com/site/revelgenomics/) were used to predict the pathogenicity of missense variants. Human Splicing Finder (http://www.umd.be/HSF), Splice AI (https://spliceailookup.broadinstitute.org/), varSEAK (https://varseak.bio/), MutationTaster, and MaxEntScan (http://genes.mit.edu/burgelab/maxent/Xmaxentscan _scoreseq.html) were applied to predict the spliceogenic effect of noncanonical variants. Variants with Splice AI score > 0.2 [10], varSEAK class > 2 (https://varseak.bio/pdf/SSP-Documentation.pdf), and the change in splice site score of MaxEntScan ≥ 15% [11] were deemed spliceogenic variants; all interpretations given by HSF and MutationTaster [12, 13], except for “No significant impact on splicing signals” or “No abrogation of potential splice sites,” were deemed to predict altered splicing.

### 2.3. Minigene Construction

Depending on the location of *MYO5B* variants, wild-type (WT) *MYO5B* exons of interest and 500-1000 bp of flanking intronic sequence were amplified by polymerase chain reaction (PCR) from a subject without an identified *MYO5B* variant using 2xTaq PCR Mix (KT201, TIANGEN BIOTECH). Specific primers, provided in Table S1, were used to introduce additional recombination sequences, and the purified WT amplicons were subcloned into the XhoI site of a modified pcDNA3.1 vector using the ClonExpress® Ultra One Step Cloning Kit (C115, Vazyme). Site-directed mutagenesis introduced c.3538-1G>A [4], c.839-1G>A [7], c.4852+11A>G [4], c.2090+3A>T [7], c.1322+5G>A [7], c.2414+5G>T [4], c.2349A>G [14], c.3045+3A>T [15], c.1669-35A>C, c.455+8T>C, c.2415-6C>G, c.613-11G>A, and c.4221G>A variants individually, using KOD-Plus-Mutagenesis Kit (SMK-101, Toyobo Bio-Technology). All mutagenesis primers are available in Table S1, and all minigenes were verified by the Sanger sequencing (Biosune, China). The workflow of the minigene protocol is outlined in Figure S2.

### 2.4. Cell Culture and Transfection

Human embryonic kidney 293T and HepG2 cells were maintained in Dulbecco's modified Eagle's medium supplemented with 10% fetal bovine serum and 1% penicillin-streptomycin at 37°C in 5% CO_2_. Cells were seeded in six-well plates and 24 h later were transiently transfected with 1 *μ*g of the WT, mutated, or empty minigenes using jetPRIME® transfection reagent, respectively (Polyplus Transfection, French).

### 2.5. RT-PCR Analysis

RNA from cells was extracted using the Direct-zol™ RNA Miniprep kits (Cat. R2050, ZYMO RESEARCH), and RNA from fresh whole peripheral blood was extracted using HiPure PX Blood RNA Kit (R4168, Magen). A total of 1 *μ*g RNA was reverse-transcribed into complementary DNA using the PrimeScript™ RT reagent kit with gDNA Eraser (Cat. RR047A, Takara). PCR was then performed with 2xTaq PCR Mix using specific primers (Table S1). PCR products were separated on 2% agarose gels to analyze product size, and the product sequences were then verified by the Sanger sequencing (Biosune, China). For mixed bands, pClone007 Simple Vector Kit (TSV-007S, Tsingke) was used to obtain the single sequence data.

### 2.6. Quantitative Real-Time PCR (qPCR)

If normal spliced transcript was identified, qPCR was performed with a QuantStudio 3 Real-Time PCR Systems (Applied Biosystems) using TB Green® Premix Ex Taq™ (Cat. RR420A, Takara). The exon-specific primers spanning the exon-exon boundaries of the *MYO5B* exon and the flanking adenovirus exons are applied and detailed in Table S1.

## 3. Results

### 3.1. Clinical Features of New Low GGT Cholestasis Patients with Biallelic *MYO5B* Variants

**Figure 1:**
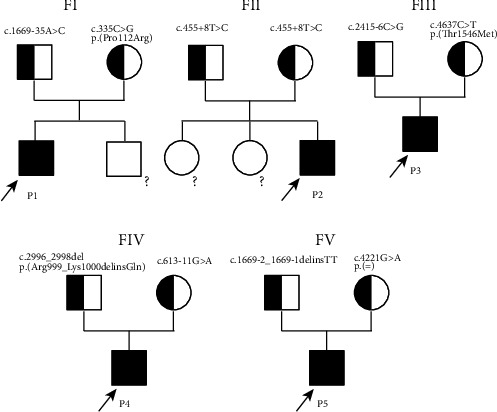
Pedigrees of 5 new low GGT cholestasis patients with biallelic *MYO5B* variants. Five patients were found to be compound heterozygous or homozygous for variants in *MYO5B* (all by exome sequencing). ?, absence of the Sanger sequencing for sample not available; F, family; P, patient.

All five new patients (Figure 1 and Table 1) were born to healthy nonconsanguineous parents. Four of them were male term babies, and P5 was a boy born prematurely at 29 weeks of gestation. All patients were suspected as having an underlying genetic etiology for low GGT cholestasis. Three patients had extrahepatic abnormalities: P3 had mild congenital heart abnormalities (atrial septal defect and patent foramen ovale) and right hydrocele; P4 had kidney abnormalities including enlargement of the kidney and *α*1-microglobinuria; P5 had hypothyroidism, severe malnutrition, and a history of self-limited diarrhea (loose stools, 4-5 times/day, and persisted for nearly 1 month at the age of 1 year and 10 months). Table 1 summarizes the clinical findings and biochemical data.

In P1, jaundice first occurred at the age of 4 days and resolved at the age of 20 days but recurred when he was 1 month old. Thereafter, conjugated hyperbilirubinemia and elevated transaminase levels appeared intermittently, but the elevation of TBA persisted during follow-up. Pruritus was observed at the age of 16 months and persisted during follow-up. Hepatomegaly, splenomegaly, and lithiasis were not observed. A needle liver biopsy at the age of 2 years and 1 month showed canalicular cholestasis and mild portal inflammation. Routine treatments, including UDCA and fat-soluble vitamin, were used, and cholestyramine, rifampicin, and phenobarbitone were added as necessary. At the last follow-up (2 years and 4 months), he continues to experience cholestatic symptoms and growth retardation.

P2 presented with jaundice at the age of 2 months. Physical examination and abdominal ultrasonography confirmed hepatomegaly. Pruritus occurred at the age of 7 months. Liver histology at the age of 1 year and 1 month showed features of cholestasis, mild portal inflammation, and slight ductular reaction. Although UDCA and antipruritic medications (cholestyramine, rifampicin, and phenobarbitone) were used, serum bilirubin, transaminase, and bile acid levels remained above normal ranges, and pruritus remained during the follow-up. Additionally, he experienced failure to thrive and growth retardation.

P3 was conceived through in vitro fertilization. He presented at 40 days of age with severe jaundice and a significant prolonged prothrombin time that was unresponsive to parenteral administration of vitamin K. He was admitted to the intensive care unit at his local hospital, where abdominal ultrasonography showed hepatomegaly and splenomegaly. Liver histology was not available. Behavior indicating pruritus was reported by his mother at the age of 2 months; however, we only found mild jaundice without scratches or ceaseless scratching at 4 months of age, when he was first referred to our clinic. He was treated with UDCA and fat-soluble vitamins, and subsequently his jaundice, hepatomegaly, splenomegaly, and all biochemical tests improved. He was free of cholestatic symptoms with all biochemical test results returned to normal at the last follow-up (2 years and 5 months). Height and weight were normal for age.

P4 presented with jaundice and severe pruritus at the age of 1 year and 7 months. He received treatment with prednisone and azathioprine for suspected autoimmune liver disease in a local hospital. During the 1.5 years of immunosuppressive treatment, the biochemical data fluctuated but remained above the normal range most of the time. He was referred to our hospital at the age of 3 years and 3 months for severe jaundice and pruritus attack following a reduced dose of prednisone. Jaundice and scratches were observed, and abdominal ultrasonography confirmed enlarged liver and kidney. Serum laboratory tests showed cholestasis with normal GGT activity and elevated transaminase levels. Kidney function tests were normal, but urine tests showed *α*1-microglobinuria. Liver histology performed on a prior biopsy (in a local hospital) showed hepatocellular and canalicular cholestasis, ballooning degeneration of hepatocytes, portal fibrosis and mild inflammation, ductular reaction, and reduction of interlobular ductules. The immunosuppressive treatment was stopped, and UDCA therapy was continued. Cholestyramine and rifampicin were also added. He continues to experience cholestatic symptoms (3 years and 4 months).

At the age of 3 months, biochemical tests performed on P5 showed cholestasis with mildly elevated bilirubin, normal GGT activity, elevated transaminase, and total bile acid levels. Pruritus was observed at the age of 1 year. Abdominal ultrasonography showed a normal liver and biliary tree. Liver histology at 1 year and 4 months showed mild portal fibrosis and inflammation and absence of ductular reaction. UDCA, cholestyramine, phenobarbitone, and fat-soluble vitamin were given. At the last follow-up (2 years), he still had persistent cholestasis despite receiving UDCA and cholestyramine therapy and experienced fluctuations in the severity of pruritus and persistent elevation in biochemical data. He suffered from severe malnutrition and poor growth. It should be noted that P5 had hypothyroidism at the first few days of life and has been continuing to receive Euthyrox therapy.

### 3.2. *In Silico* Prediction of *MYO5B* Variants

In the 5 new patients, 9 novel *MYO5B* potential pathogenic variants were identified, including 4 noncanonical splice site intronic variants, 2 missense variants, 1 synonymous variant, 1 canonical splice site variant, and 1 in-frame deletion. Literature searching yielded 6 *MYO5B* noncanonical variants [4, 7, 14, 15]. The *in silico* prediction and allele frequency of the 11 *MYO5B* noncanonical variants are detailed in Table 2. The *in silico* prediction and/or allele frequency of other novel variants are described as follows: variant c.335C>G/p.(Pro112Arg) has never been reported in gnomAD, predicted pathogenic by five above mentioned *in silico* tools; variant c.4637C>T/p.(Thr1546Met) had an allele frequency of 0/135/280948 (the number of homozygotes/heterozygotes/total persons tested) in gnomAD, predicted pathogenic by 2 (Polyphen2 and MutationTaster) out of the 5 *in silico* tools; c.2996_2998del/p.(Arg999_Lys1000delinsGln) and c.1669-2_1669-1delinsTT have never been reported in gnomAD.

### 3.3. Splicing Effects of *MYO5B* Variants in Minigene System

**Figure 2:**
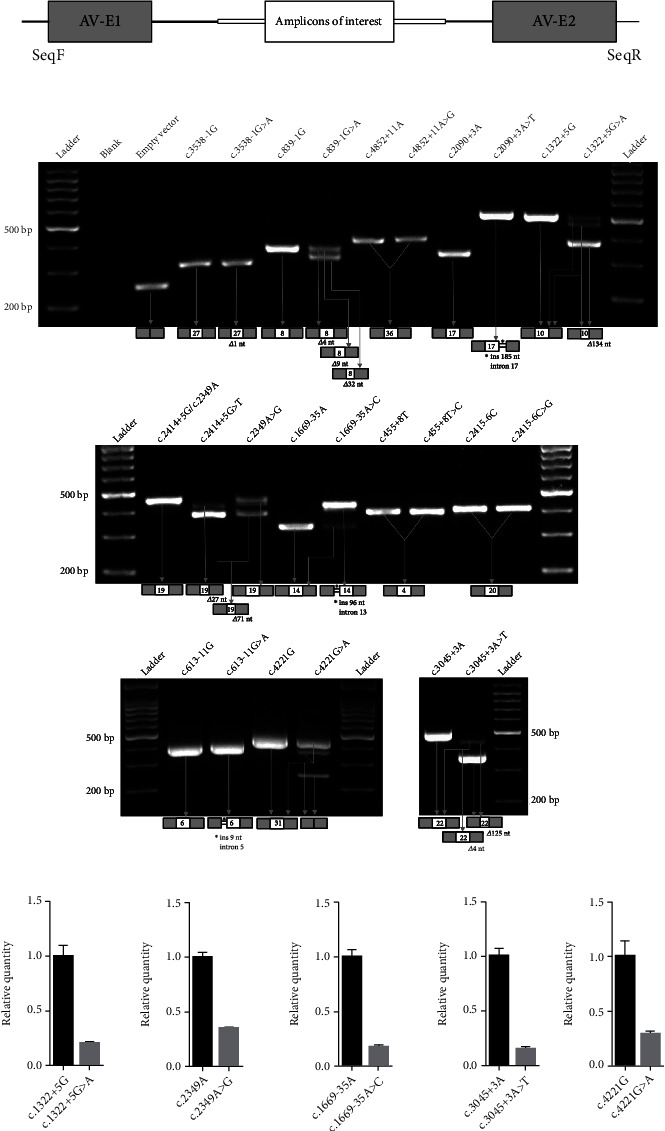
Splicing effects of *MYO5B* variants based on minigene splicing assays. (a) Schematic of minigene system. AV-E1 and AV-E2 are adenovirus exon 1 and exon 2, embedded in the pcDNA3.1 vector; amplicons of interest (exons of interest and 500~1000 bp of flanking intronic sequences in this study) were inserted into the pcDNA3.1 vector. SeqF and SeqR are primers for cDNA amplifying and the Sanger sequencing. (b) The splicing products were amplified by PCR and underwent 2% agarose gel electrophoresis. Gray boxes indicate two adenovirus exons; white boxes are *MYO5B* exons and are marked with an exon number individually; the arrangement of boxes is consistent with the schematic of minigene system. c.3538-1G>A and c.839-1G>A were selected as positive controls for the assay. *Δ*, deletion; ins, insertion; nt, nucleotides; bp, base pairs. (c) qPCR for relative quantity of the normal splicing in the mutant minigenes with correctly spliced transcript identified. Compared to the wild-type minigenes, c.1322+5G>A, c.2349A>G, c.1669-35A>C, c.3045+3A>T, and c.4221G>A have only 20%, 35%, 17%, 15%, and 30% of normal splicing, respectively. The first variant in each pair represents the common allele, while the second variant represents the rare allele.

As shown in Figures 2(a) and 2(b) and Figure S3, all *MYO5B* WT alleles resulted in normal splicing, and two canonical splice site variants (c.3538-1G>A and c.839-1G>A) resulted in pre-mRNA splicing errors as expected, indicating that the minigene RNA splicing assays could accurately evaluate pre-mRNA splicing. Of the 11 analyzed variants, 3 (c.4852+11A>G, c.455+8T>C, and c.2415-6C>G) produced normal slicing, and the other 8 resulted, to some extent, in aberrant splicing.

The splicing alterations of 8 variants could be subdivided into two groups. The first group consisted of 3 variants, including c.2090+3A>T, c.2414+5G>T, and c.613-11G>A, which led to complete splicing aberrations. Variant c.2090+3A>T caused retention of 185 nucleotides from intron 17 and predicted to encode a truncated protein, MYO5B p.Trp698Leufs^∗^48; c.2414+5G>T caused the deletion of the last 71 and 27 nucleotides of exon 19, respectively, by activating a preexisting cryptic 5′ splice site within the exon and predicted to encode a truncated protein, MYO5B p.Val782Alafs^∗^3 (major product), as well as a protein with 12 amino acids deletion (p.Tyr797_Arg805del); c.613-11G>A caused retention of 9 nucleotides from intron 5 and predicted to encode a protein with 3 amino acids insertion (p.Glu204_Ala205insSerValGln).

In the second group of 5 variants (c.1322+5G>A, c.2349A>G (p.=), c.1669-35A>C, c.3045+3A>T, and c.4221G>A (p.=)), aberrant pre-mRNA splicing and partially normal splicing were simultaneously present. The c.1322+5G>A caused a prominent deletion of the last 134 nucleotides of exon 10 by decreasing the strength of the reference splice site and simultaneously activating a preexisting cryptic 5′ splice site within the exon and predicted to encode a truncated protein, MYO5B p.Ile398^∗^, which was the major product, and the minor product corresponded to the correct splicing with a mean percentage of 20% WT splicing. Variant c.2349A>G showed two bands: one causing the deletion of the last 71 nucleotides of exon 19 and the second corresponding to the normal splicing with a mean percentage of 35% WT splicing. Variant c.1669-35A>C resulted in predominant retention of 96 nucleotides from intron 13, which was predicted to encode a truncated protein, MYO5B p.Val557Leufs^∗^11, and also, a small percentage of the normally spliced product (with a mean percentage of 17% WT splicing) was detected. c.3045+3A>T caused a prominent deletion of the last 125 nucleotides of exon 22 by activating a preexisting cryptic 5′ splice site within the exon and predicted to encode a truncated protein, MYO5B p.Gly974Alafs^∗^15; a small percentage of products revealed a deletion of the last 4 nucleotides of exon 22 (also expected to cause a premature stop codon) and the normal splicing (with a mean percentage of 10% WT splicing). Variant c.4221G>A showed two bands: one causing the exon 31 skipping and the second corresponding to the normal splicing with a mean percentage of 30% WT splicing. The qPCR results are shown in Figure 2(c), and the possible consequences of aberrant splicing (mRNA changes and predicted protein changes) in the analyzed *MYO5B* variants were summarized in Figure S4.

### 3.4. Blood RNA Analysis *In Vivo*

Fresh peripheral blood samples from P4 and his family members were available for further analysis. RNA was extracted and RT-PCR was performed. The Sanger sequencing showed that P4 and his mother (who carried c.613-11G>A) had two transcripts: one with 9 nucleotides intron 5 retention and another normal transcript, whereas his father (who did not carry c.613-11G>A and served as a normal control) only had the normal transcript (Figure S5). The splicing change observed was consistent with the result obtained from the minigene assay.

### 3.5. ACMG Reclassification

Supplemented with the functional analysis, the pathogenicity of noncanonical variants was reevaluated according to the ACMG guidelines [8, 9]. Eight previous VUSs (c.2349A>G/p.(=), c.4221G>A/p.(=), c.613-11G>A, c.1322+5G>A, c.1669-35A>C, c.2090+3A>T, c.2414+5G>T, and c.3045+3A>T) were reclassified as likely pathogenic, and three (c.4852+11A>G, c.455+8T>C, and c.2415-6C>G) were reclassified as likely benign (Table 2).

## 4. Discussion

With the increasing use of next-generation sequencing in clinical practice, including targeted panel sequencing, WES, and even whole-genome sequencing, VUS reporting has become more common. This poses a challenge for clinical management and genetic counseling; for example, a definitive genetic diagnosis cannot be made for some patients with low GGT cholestasis, and biallelic *MYO5B* VUSs identified due to caution should be exercised in clinical diagnostics regarding VUSs. The reported cases of *MYO5B*-associated FIC are limited, and a small portion of variants is VUSs. However, variants can be reclassified over time based on functional studies.

Canonical splicing variants are typically located in intronic splicing donor/acceptor sites and often disrupt pre-mRNA splicing, whereas variants outside these sites are frequently classified as benign or VUSs [16]. In addition, synonymous variants are more challenging to determine their pathogenicity than nonsynonymous single nucleotide variants because they do not alter the protein sequence. Several studies have shown that synonymous and intronic variants can also affect gene function by disrupting normal pre-mRNA splicing [17–20]. Therefore, splicing pattern analysis can help reduce uncertainty in clinical pathogenicity interpretation. Minigene splicing assays are widely used in splicing analysis when RNA samples from patients are not available, and their results have been found to closely resemble endogenous splicing patterns identified from patient RNA [21, 22]. Therefore, this study focuses on performing minigene splicing assays and patient-derived RNA analysis on noncanonical variants in *MYO5B* obtained from a literature review and retrospective clinical collection rather than on more obviously pathogenic variants.

Several noncanonical variants in *MYO5B* have been associated with low GGT cholestasis in recent years [4, 7, 14, 15]; meanwhile, some new potential pathogenic *MYO5B* noncanonical variants have also been clinically detected. Although some *in silico* tools predict them to be pathogenic, the precise effect of these variants on pre-mRNA splicing has not been experimentally determined, except for c.2090+3A>T, which has been analyzed using minigene splicing assays in a recently published study while our experiments were ongoing, and our results are consistent with those reported in the literature [23]. Herein, we investigated eleven *MYO5B* noncanonical variants, including 6 reported and 5 novel ones. Minigene splicing assays revealed that 8 out of 11 variants, including 5 reported variants (c.2349A>G/p.(=), c.1322+5G>A, c.2090+3A>T, c.2414+5G>T, and c.3045+3A>T) and 3 newly identified variants (c.4221G>A/p.(=), c.613-11G>A, and c.1669-35A>C), disrupted pre-mRNA splicing. RNA samples obtained from P4 with c.613-11G>A were also subjected to RT-PCR and sequencing analysis, and the results were consistent with the minigene assay. The variants c.4852+11A>G, c.455+8T>C, and c.2415-6C>G were not found to significantly affect splicing based on minigene validation. These findings can aid in the reclassification of these variants and contribute to more precise clinical management.

The pathogenicity of a variant depends on both the clinical setting and functional evidence. Based on the functional analysis and following the ACMG guidelines, the biallelic *MYO5B* variants found in three new patients (P1, P4, and P5) can be classified as likely pathogenic, and therefore, these patients can be genetically diagnosed as *MYO5B-*associated FIC. However, the diagnosis of a previously reported *MYO5B*-associated FIC patient [4] may need to be redefined, as the variant c.4852+11A>G has been confirmed not to affect splicing in this study. The results show the ability of RNA splicing analysis to clarify variant interpretation, as abnormal splicing can provide supporting evidence of pathogenicity. It should be noted that the novel variants c.1669-35A>C (carried by P1) and c.613-11G>A (carried by P4), which are located 35 bp and 11 bp from canonical splice site, respectively, would not typically be suspected or classified as pathogenic without further functional studies. Minigene analysis and blood RNA analysis showed these two variants resulted in aberrantly spliced transcripts, and thus, they can be reclassified as likely pathogenic. The novel variants c.335C>G/p.(Pro112Arg) (carried by P1) and c.2996_2998del/p.(Arg999_Lys1000delinsGln) (carried by P4) can also be reclassified as likely pathogenic based on the evidence of a likely pathogenic variant detected in trans. We therefore suggest that functional studies should be introduced to intronic spliceogenic variants if *MYO5B*-associated FIC is strongly suspected; it has been reported that many deep intronic variants can disrupt splicing and thus can become a cause of genetic disorders [20, 24, 25]. This study also showed that about 73% of the selected *MYO5B* noncanonical variants can result in aberrantly spliced transcripts.

The definitive diagnosis for the other two patients (P2 and P3) could not be established based on the current evidence. The variants they carry, c.455+8T>C and c.2415-6C>G, respectively, have no effect on splicing and are now being reclassified as likely benign, which means that they cannot be considered as causative variants. Further investigations, such as liver RNA-seq of the patients [26] and new disease-causing gene analysis, may make a difference.

*In silico* splice prediction tools have been developed to estimate the likelihood of a noncanonical variant being pathogenic, but they are likely making errors, and it has been reported that the choice of the best splice prediction tool may depend on the gene and the type of splice-altering variant [19, 27, 28]. To assess the performance of individual *in silico* prediction tool, we calculated the accuracy, positive predictive value, and negative predictive value in Table S2. Among the eight variants confirmed to affect splicing, HSF accurately identified all of them, while Splice AI correctly identified seven out of the eight as affecting splicing. Regarding the three variants confirmed to have no effect on splicing, Splice AI correctly identified all of them, with HSF accurately predicted two out of the three variants. There was a tendency for the variants found experimentally to not affect splicing to be more frequently predicted as such. The accuracy of HSF and Splice AI was relatively high at approximately 91%. HSF demonstrated a perfect negative predictive value, while Splice AI achieved a perfect positive predictive value. In contrast, the performance of MutationTaster as a splicing prediction tool is extremely poor, with an accuracy of approximately 45%. Our study's results partially align with the previous large-scale analyses that showed an accuracy of approximately 91%, a positive predictive value of approximately 84%, and a negative predictive value of around 95% for Splice AI. For HSF, the accuracy was around 58%, with a lower positive predictive value but a higher negative predictive value of approximately 86% [20]. Based on these factors, we recommend utilizing Splice AI and HSF for splice prediction. Our results that suggest *in silico* tools could assist in the clinical interpretation of variants potentially affecting splicing, but this method cannot substitute for functional analysis since experimental evidence of abnormal splicing is more reliable than splicing predictions according to the guidelines of ACMG. Hence, the incorporation of *in silico* splicing prediction and experimental splicing analysis may be helpful to ensure accurate clinical interpretation of VUSs. In addition, we observed that two variants (c.455+8T>C and c.2415-6C>G) did not impact pre-mRNA splicing. Although these variants had frequencies below 0.001 in the overall population, they exhibited higher frequencies in the East Asian population, all exceeding 0.001. Assessing the allele frequency of a variant is important when determining whether it is “too common” or “sufficiently rare” for a Mendelian disease. Consequently, we would recommend using as a cutoff the highest frequency seen in any population, rather than the overall frequency in the database.

In conclusion, we have characterized the molecular consequences of 11 *MYO5B* noncanonical variants, which represent all known *MYO5B* noncanonical variants with potential pathogenic significance to date. Our functional study found that 8 of these variants resulted in aberrantly spliced transcript, thus contributing to the accurate clinical classification of these variants and improving genetic diagnosis for individuals.

## Figures and Tables

**Table 1:** Clinical findings and identified *MYO5B* variants in new low GGT cholestasis patients.

	Patient 1	Patient 2	Patient 3	Patient 4	Patient 5
Variants	c.335C>G/p.(Pro112Arg) c.1669-35A>C	c.455+8T>C	c.4637C>T/p.(Thr1546Met) c.2415-6C>G	c.613-11G>A c.2996_2998del/p.(Arg999_Lys1000delinsGln)	c.4221G>A/p.(=) c.1669-2_1669-1delinsTT
Zygosity	Compound heterozygote	Homozygote	Compound heterozygote	Compound heterozygote	Compound heterozygote
Sex/age at presentation	Male/1 m	Male/2 m	Male/1 m 10 d	Male/1y 7 m	Male/3 m
Liver manifestations	Jaundice, pruritus	Jaundice, pruritus, hepatomegaly	Jaundice, pruritus, hepatomegaly, splenomegaly, liver failure	Jaundice, pruritus, hepatomegaly	Jaundice, pruritus, hepatomegaly
Liver biopsy age/findings	2 y 1 m/canalicular cholestasis, mild portal inflammation	1 y 1 m/canalicular cholestasis, mild portal inflammation, slight ductular reaction	ND	1 y 8 m/hepatocellular and canalicular cholestasis, ballooning degeneration of hepatocytes, portal fibrosis and mild inflammation, ductular reaction, and reduction of interlobular ductules	1 y 4 m/mild portal fibrosis and inflammation and features suggestive of chronic cholestasis
Extrahepatic abnormalities	None	None	Right hydrocele, atrial septal defect, and patent foramen ovale	Enlargement of the kidney and *α*1-microglobinuria	Hypothyroidism, severe malnutrition, premature, and diarrhea
Management	UDCA, rifampicin, cholestyramine, phenobarbitone, fat-soluble vitamins	UDCA, rifampicin, cholestyramine, phenobarbitone, fat-soluble vitamins	UDCA, fat-soluble vitamins	UDCA, rifampicin, cholestyramine, fat-soluble vitamins	UDCA, cholestyramine, phenobarbitone, fat-soluble vitamins, Euthyrox
Status at first assessment					
Age	4 m	4 m	2 m 23 d	1 y 7 m	3 m
Pruritus	–	–	–	+	–
Hepatomegaly	–	+	+	+	–
Splenomegaly	–	–	+	–	–
TB (3.4-17.1 *μ*mol/L)	112.6	264	274.8	227.7	30
DB (0-6 *μ*mol/L)	79.2	231.4	196.2	157.8	26
ALT (9-50 IU/L)	517	40.3	299	156	479
AST (15-40 IU/L)	572	61.7	196	114	187
GGT (8-57 IU/L)	46	27.9	54	13	47
TBA (0-10 *μ*mol/L)	101.8	141.5	165.7	436.7	109
ALB (40-55 g/L)	43.6	40.5	41.8	43.4	44
PT (11-14.5 s)^†^	ND	12.3	43	ND	ND
Status at most recent assessment					
Age	2 y 4 m	2 y 5 m	2 y 5 m	3 y 4 m	2 y
Pruritus	+	+	–	+	+
Hepatomegaly	–	+	–	+	–
Splenomegaly	–	–	–	–	–
TB (3.4-17.1 *μ*mol/L)	94.8	114.1	5	104.1	215.3
DB (0-6 *μ*mol/L)	84.6	88.6	2.1	77	163.8
ALT (9-50 IU/L)	37	35.3	25.37	39.88	171.12
AST (15-40 IU/L)	68	65.2	43.53	59.73	125.57
GGT (8-57 IU/L)	8	18	7.8	7.95	11.32
TBA (0-10 *μ*mol/L)	290.2	185.5	3.9	103	380.80
ALB (40-55 g/L)	44.5	37.5	43.4	39.37	34.74
PT (11-14.5 s)^†^	12.0	12.2	13.8	11	12.2
Weight centile	3^rd^-10^th^	<3^rd^	75^th^	25^th^-50^th^	<3^rd^
Height centile	<3^rd^	<3^rd^	90^th^	25^th^-50^th^	<3^rd^
Outcome	Mild jaundice, pruritus, poor growth	Mild jaundice, pruritus, poor growth	Atrial septal defect	Jaundice and pruritus	Jaundice, pruritus, poor growth

Normal ranges are given in parentheses. Abbreviations: ALT, alanine aminotransferase; AST, aspartate aminotransferase; DB, directed bilirubin; d, day(s); GGT, gamma-glutamyltransferase; m, month(s); NA, not available; ND, not done; PT, prothrombin time; s, seconds; TB, total bilirubin; TBA, total bile acids; y, year(s); +, present; –, absent. ^†^Values after supplementation of vitamin K.

**Table 2:** *In silico* and functional analysis for *MYO5B* intronic and synonymous variants.

Variants	Location	Human splicing finder	Splice AI (score)	varSEAK (class)	MutationTaster	MaxEntScan	Minigene splicing assay	Blood RNA analysis	Allele frequency in gnomAD (hom/het/total)^†^	Original classification	Reclassification
**c.1669-35A>C**	Intron 13	Alteration of the WT branch point may affect splicing	Acceptor loss (0.14)	No splicing effect (1)	No abrogation of potential splice sites	6.33⇒6.33	Inserting 96 nt of intron 13/normal splicing (~17%)	NA	—	VUS (PM2_P+PP3+PP4)	Likely pathogenic (PM2_P+PP4+PS3)

**c.455+8T>C**	Intron 4	No significant impact on splicing signals	No splicing effect (0)	No splicing effect (1)	Donor increased	9.22⇒9.22	No splicing effect	NA	0/71/280946	VUS (PM2_P+PM3_P+PP3+PP4)	Likely benign (PM3_P+PP4++BS3)

**c.2415-6C>G**	Intron 19	Alteration of the WT acceptor site, most probably affecting splicing	No splicing effect (0)	Likely no splicing effect (2)	Acceptor gained	10.21⇒6.81 (-33.3%)	No splicing effect	NA	0/169/279700	VUS (PM2_P+PP3)	Likely benign (PP3+BS3)

**c.613-11G>A**	Intron 5	Alteration of the WT acceptor site, most probably affecting splicing	Acceptor loss (0.84) Acceptor gain (0.99)	Use of de novo splice site 9 nt upstream of 3′ splice site and loss of function for authentic splice site (5)	Donor increased Donor gained	9.06⇒3.19 (-64.8%)	Inserting 9 nt of intron 5	Inserting 9 nt of intron 5	—	VUS (PM2_P+PP3+PP4)	Likely pathogenic (PM2_P+PP3+PP4+PS3)

**c.4221G>A/p.(=)**	Exon 31	Alteration of the WT donor site, most probably affecting splicing	Donor loss (0.32)	Loss of function for authentic splice site, exon skipping (5)	Alteration within used splice site, likely to disturb normal splicing	6.29⇒1.46 (-76.8%)	Exon 31 skipping/normal splicing (~30%)	NA	—	VUS (PM2_P+PM3+PP3+PP4)	Likely pathogenic (PM2_P+PM3+PP3+PP4+PS3)

c.4852+11A>G	Intron 36	No significant impact on splicing signals	No splicing effect (0)	No splicing effect (1)	No abrogation of potential splice sites	8.89⇒8.89	No splicing effect	NA	—	VUS (PM2_P+PM3_P+PP4)	Likely benign (PM2_P+PM3_P+PP4+BS3)

c.2090+3A>T	Intron 17	Alteration of the WT donor site, most probably affecting splicing	Donor loss (0.85)	Loss of function for authentic splice site, strong decrease of score for authentic splice site, exon skipping (5)	Donor lost	8.63⇒0.49 (-94.3%)	Inserting 185 nt of intron 17	NA	—	VUS (PM2_P+PM3_P+PP3+PP4)	Likely pathogenic (PM2_P+PM3_P+PP3+PP4+PS3)

c.1322+5G>A	Intron 10	Alteration of the WT donor site, most probably affecting splicing	Donor loss (0.28)	Loss of function for authentic splice site, strong decrease of score for authentic splice site, exon skipping (5)	No abrogation of potential splice sites	6.03⇒-0.42 (-107%)	134 nt deletion of exon10/normal splicing (~20%)	NA	—	VUS (PM2_P+PM3+PP3+PP4)	Likely pathogenic (PM2_P+PM3+PP3+PP4+PS3)

c.2414+5G>T	Intron 19	Alteration of the WT donor site, most probably affecting splicing	Donor loss (0.67) Donor gain (0.42)	Strong decrease of score for authentic splice site, use of a cryptic site 27 nt upstream of 5′ splice site (5)	No abrogation of potential splice sites	9.60⇒4.28 (-55.4%)	71 nt and 27 nt deletions of exon 19, respectively	NA	—	VUS (PM2_P+PM3+PP3+PP4)	Likely pathogenic (PM2_P+PM3+PP3+PP4+PS3)

c.2349A>G/p.(=)	Exon 19	Activation of a cryptic donor site, potential alteration of splicing	Donor loss (0.09) Donor gain (0.25)	No splicing effect (1)	No abrogation of potential splice sites	9.60⇒9.60	71 nt deletion of exon 19/normal splicing (~35%)	NA	0/1/249462	VUS (PM2_P+PM3+PP3+PP4)	Likely pathogenic (PM2_P+PM3+PP3+PP4+PS3)

c.3045+3A>T	Intron 22	Alteration of the WT donor site, most probably affecting splicing	Donor loss (0.52)	Loss of function for authentic splice site, strong decrease of score for authentic splice site, exon skipping (5)	Donor lost	9.55⇒5.42 (-43.2%)	125 nt and 4 nt deletion of exon 22, respectively/normal splicing (~10%)	NA	—	VUS (PM2_P+PM3_P+PP3+PP4)	Likely pathogenic (PM2_P+PM3_P+PP3+PP4+PS3)

Abbreviations: NA, not applicable; —, not reported; nt, nucleotides; VUS, Variant of uncertain significance; PM2_P, PM2_Supporting; PM3_P, PM3_Supporting. PM2: absent from controls (or at extremely low frequency if recessive) in Exome Sequencing Project; PM3: for recessive disorders, detected in trans with a pathogenic or likely pathogenic variant; PP3: multiple lines of computational evidence support a deleterious effect on the gene or gene product (conservation, evolutionary, splicing impact, etc.); PP4: patient's phenotype or family history is highly specific for a disease with a single genetic etiology; PS3: well-established in vitro or in vivo functional studies supportive of a damaging effect on the gene or gene product; BS3: well-established in vitro or in vivo functional studies show no damaging effect on protein function or splicing. ^†^The number of homozygotes/heterozygotes/total in population. Variants in boldface indicate novel.

## Data Availability

All data relevant to the study are included in the article or the supplementary materials attached.

## Abbreviations

bp: Base pair
FIC: Familial intrahepatic cholestasis
GGT: Gamma-glutamyltransferase
MYO5B: Myosin 5B
MVID: Microvillus inclusion disease
pre-mRNA: Premessenger RNA
UDCA: Ursodeoxycholic acid
VUSs: Variants of uncertain significance
WES: Whole-exome sequencing.

## Supplementary Materials

Supplementary 1 Figure S1: the flowchart of patient inclusion.

Supplementary 2 Figure S2: the workflow of the minigene protocol.

Supplementary 3 Figure S3: the Sanger sequencing data of transcripts identified in minigenes.

Supplementary 4 Figure S4: scheme of the possible consequences of aberrant splicing in the analyzed *MYO5B* variants. Variant names are listed on the left; corresponding mRNA structures are located next to each variant name; the possible consequences of aberrant splicing are listed below each mRNA structure. The purple lines correspond to the locations of nucleotides deletion; the magenta arrows correspond to the locations of nucleotides insertion. ATG, reference start codon; TGA in black, reference stop codon; TGA in gray, disrupted reference stop codon; aa, amino acids; E, exon; nt, nucleotides; del, deletion; ins, insertion.

Supplementary 5 Figure S5: the splicing analysis of c.613-11G>A from the family IV based on blood RNA extraction. Variant c.613-11G>A was inherited from the mother.

Supplementary 6 Table S1: primers used in this research.

Supplementary 7 Table S2: performance assessment of *in silico* prediction tools on experimentally validated variants.
