# Supplementary figures and images for: Splicing Analysis of MYO5B Noncanonical Variants in Patients with Low Gamma-Glutamyltransferase Cholestasis
Source: Hum Mutat. 2023 Jul 27;2023:8848362. doi: 10.1155/2023/8848362 (PMC11918961; doi:10.1155/2023/8848362)

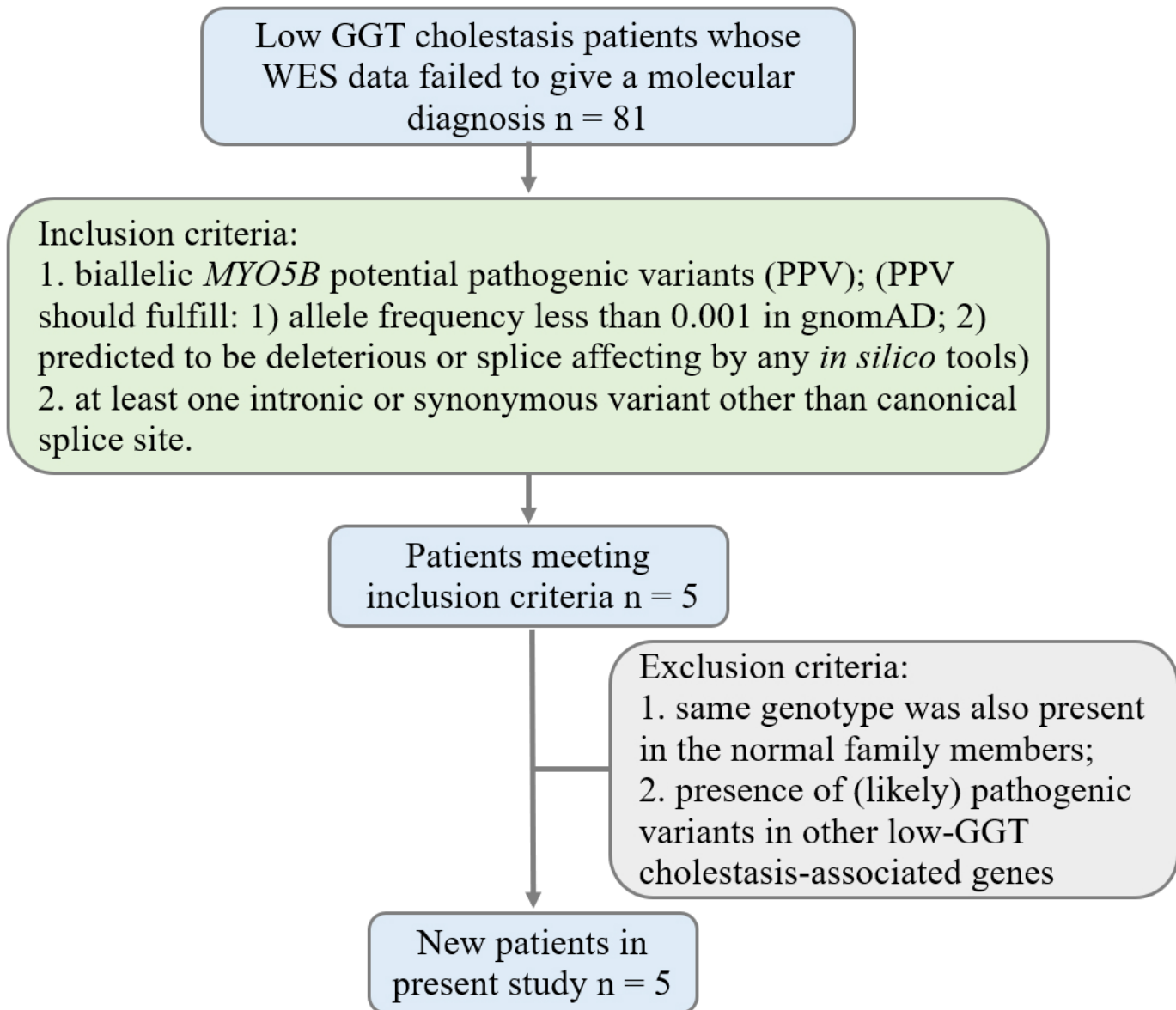

**Figure S1. The flowchart of patient inclusion**

Supplement: Supplementary 1 — Figure S1: the flowchart of patient inclusion. [file 8848362.f1.pdf]

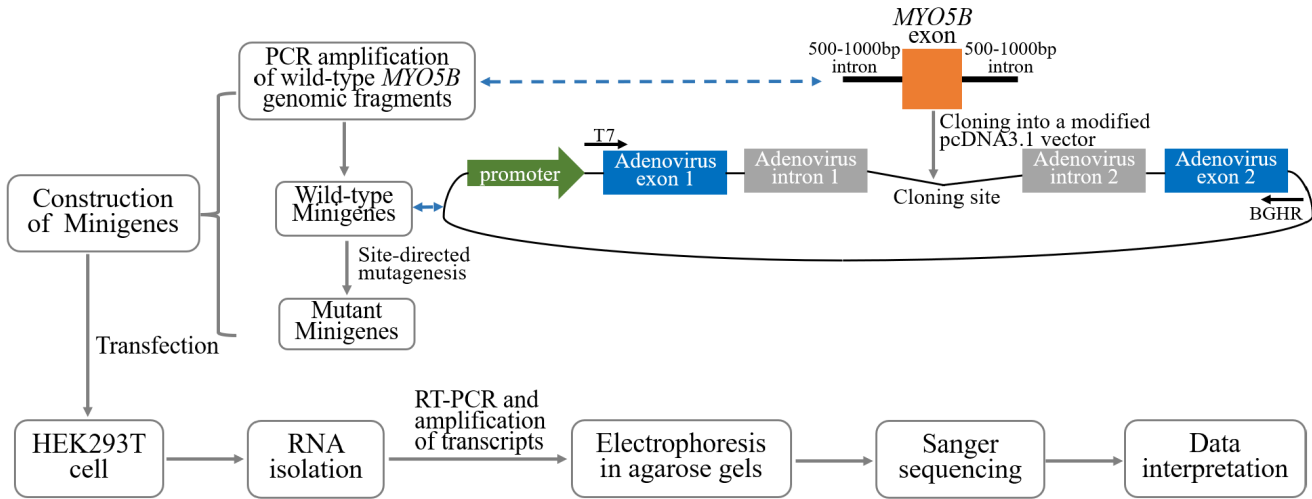

**Figure S2. The workflow of the minigene protocol.**

Supplement: Supplementary 2 — Figure S2: the workflow of the minigene protocol. [file 8848362.f2.pdf]
