# Supplementary material for: Splicing Analysis of MYO5B Noncanonical Variants in Patients with Low Gamma-Glutamyltransferase Cholestasis
Source: Hum Mutat. 2023 Jul 27;2023:8848362. doi: 10.1155/2023/8848362 (PMC11918961; doi:10.1155/2023/8848362)
Supplement: Supplementary 4 — Figure S4: scheme of the possible consequences of aberrant splicing in the analyzed MYO5B variants. Variant names are listed on the left; corresponding mRNA structures are located next to each variant name; the possible consequences of aberrant splicing are listed below each mRNA structure. The purple lines correspond to the locations of nucleotides deletion; the magenta arrows correspond to the locations of nucleotides insertion. ATG, reference start codon; TGA in black, reference stop codon; TGA in gray, disrupted reference stop codon; aa, amino acids; E, exon; nt, nucleotides; del, deletion; ins, insertion. [file 8848362.f4.pdf]

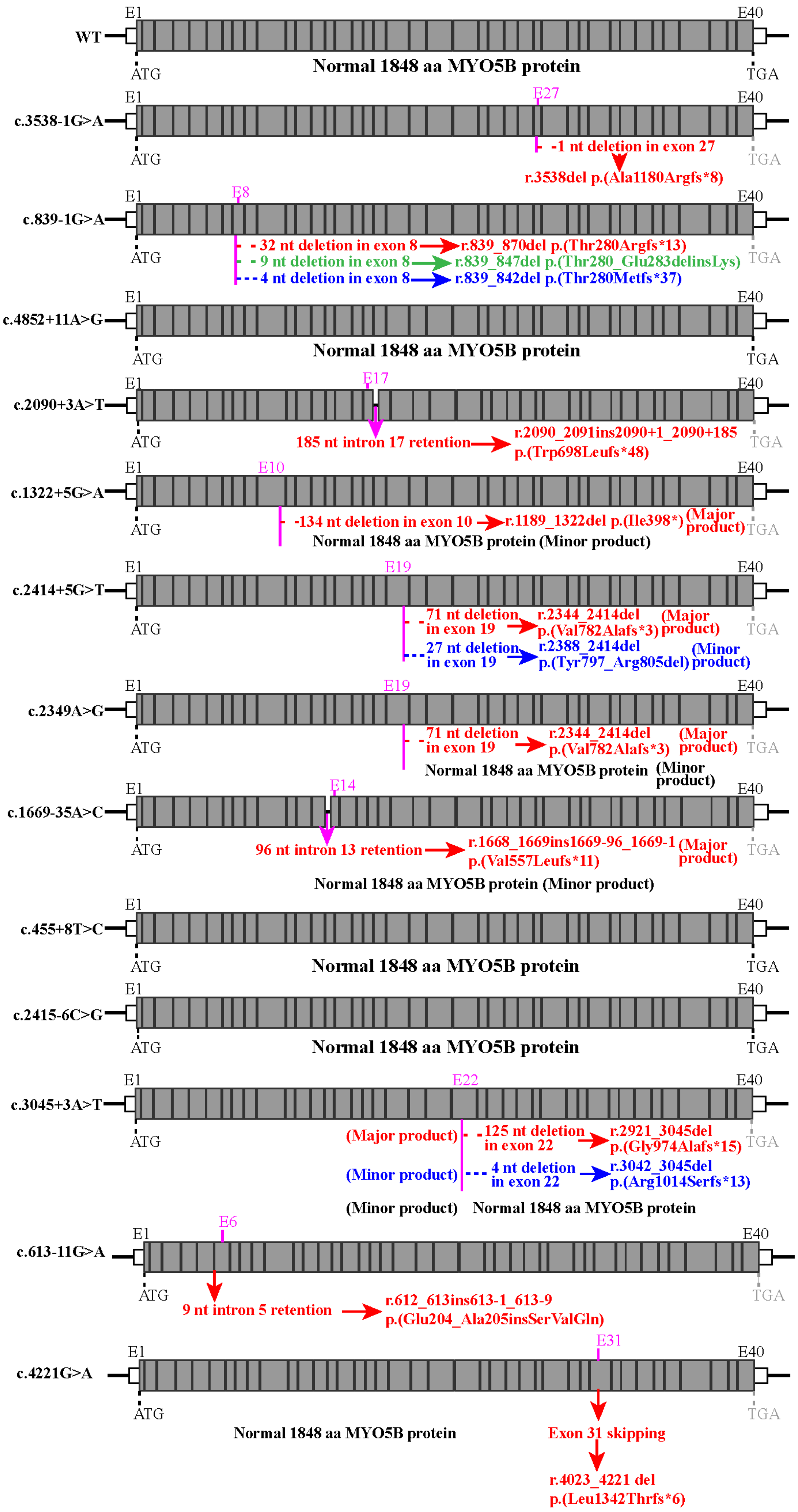

Figure S4. Scheme of the possible consequences of aberrant splicing in the analyzed *MYO5B* variants.

Variant names are listed on the left; corresponding mRNA structures are located next to each variant name; the possible consequences of aberrant splicing are listed below each mRNA structure. The purple lines correspond to the locations of nucleotides deletion; the magenta arrows correspond to the locations of nucleotides insertion. ATG, reference start codon; TGA in black, reference stop codon; TGA in gray, disrupted reference stop codon; aa, amino acids; E, exon; nt, nucleotides; del, deletion; ins, insertion.
