# Supplementary material for: Splicing Analysis of MYO5B Noncanonical Variants in Patients with Low Gamma-Glutamyltransferase Cholestasis
Source: Hum Mutat. 2023 Jul 27;2023:8848362. doi: 10.1155/2023/8848362 (PMC11918961; doi:10.1155/2023/8848362)
Supplement: Supplementary 5 — Figure S5: the splicing analysis of c.613-11G>A from the family IV based on blood RNA extraction. Variant c.613-11G>A was inherited from the mother. [file 8848362.f5.pdf]

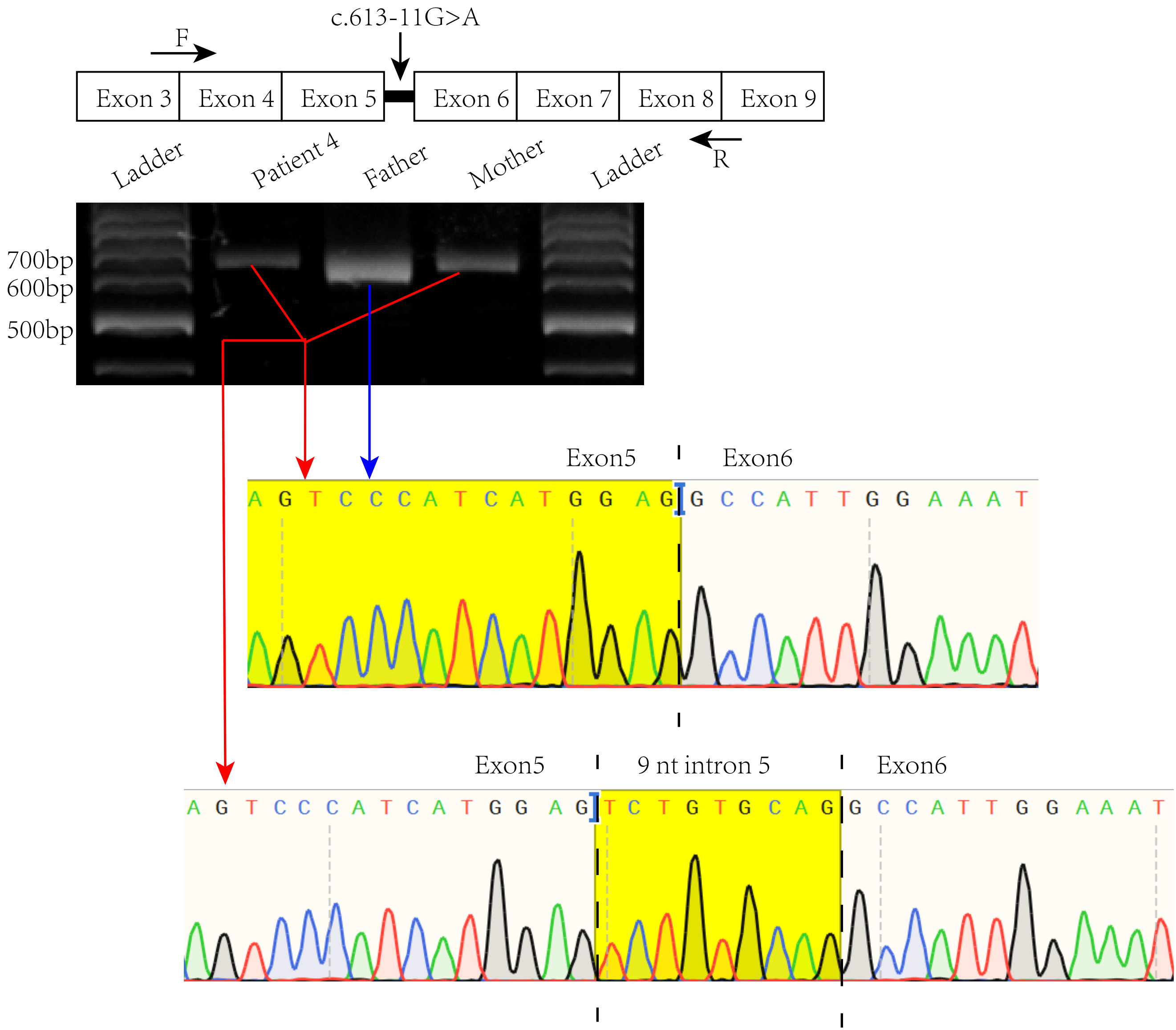

Figure S5. The splicing analysis of c.613-11G>A from the family IV based on blood RNA extraction.

Variant c.613-11G>A was inherited from the mother.
