# Supplementary material for: Splicing Analysis of MYO5B Noncanonical Variants in Patients with Low Gamma-Glutamyltransferase Cholestasis
Source: Hum Mutat. 2023 Jul 27;2023:8848362. doi: 10.1155/2023/8848362 (PMC11918961; doi:10.1155/2023/8848362)
Supplement: Supplementary 6 — Table S1: primers used in this research. [file 8848362.f6.docx]

| **Table S1. Primers used in this research** | | |
| --- | --- | --- |
| **Cloning primers** | | |
| Name of primer | Sequence (5’-3’) | |
| 3538-F-XhoI | GTCAGCTAGCTTGGTCATCTCGAGTAATCAGGGACTAACACCCACT | |
| 3538-R-XhoI | AGGGCCCAGTTAGTACGCCCTCGAGAGCCATGGGAATGATTCAAGACT | |
| 839-F-XhoI | GTCAGCTAGCTTGGTCATCTCGAGAGGCAGGATAGTACCAGGCA | |
| 839-R-XhoI | AGGGCCCAGTTAGTACGCCCTCGAGGCCCCTGAAGGACAGAGAACT | |
| 4852-F-XhoI | GTCAGCTAGCTTGGTCATCTCGAGTTCTCTGGGTCAAGAGCTGAAC | |
| 4852-R-XhoI | AGGGCCCAGTTAGTACGCCCTCGAGAAGAGCTTCATTGGTCCGTCAT | |
| 2090-F-XhoI | GTCAGCTAGCTTGGTCATCTCGAGACCGCATCCAGCCCTTCTTATTAGT | |
| 2090-R-XhoI | AGGGCCCAGTTAGTACGCCCTCGAGGGCACAGGCATGTTAATAGTAGCAG | |
| 1322-F-XhoI | GTCAGCTAGCTTGGTCATCTCGAGGGCTAAGAAGAAAGCGAGCCT | |
| 1322-R-XhoI | AGGGCCCAGTTAGTACGCCCTCGAGGTGTCACTGTTCTTACGACGC | |
| 2414-F-XhoI | GTCAGCTAGCTTGGTCATCTCGAGCATCCTGGCTCTCCTGATCCTCAG | |
| 2414-R-XhoI | AGGGCCCAGTTAGTACGCCCTCGAGTTCTGCTCGGGATGCATGGAATGT | |
| 1669-F-XhoI | GTCAGCTAGCTTGGTCATCTCGAGAGTTAACAGTCTACCCTCATGG | |
| 1669-R-XhoI | AGGGCCCAGTTAGTACGCCCTCGAGGTGTTCCTTAAATCCCACCT | |
| 455-F-XhoI | GTCAGCTAGCTTGGTCATCTCGAGGTAAATTGTATTCTCTGTCCGC | |
| 455-R-XhoI | AGGGCCCAGTTAGTACGCCCTCGAGACTATGCACAGCTGCCAGTT | |
| 2415-F-XhoI | GTCAGCTAGCTTGGTCATCTCGAGAACAGAGATTGGTCTGGGCCTG | |
| 2415-R-XhoI | AGGGCCCAGTTAGTACGCCCTCGAGCAACTCAGGTATCAGAAGGGGT | |
| 3045-F- XhoI | GTCAGCTAGCTTGGTCATCTCGAGCTTATCGGTGATTTGGCCGC | |
| 3045-R- XhoI  613-F-XhoI  613-R-XhoI  4221-F-XhoI  4221-R-XhoI | AGGGCCCAGTTAGTACGCCCTCGAGCCACTGCAACTCCCCATTGA  GTCAGCTAGCTTGGTCATCTCGAGTCCTCTATACTCGGAGGGTGG  AGGGCCCAGTTAGTACGCCCTCGAGCCTGAGCGAAGAGAGCTAAGG  GTCAGCTAGCTTGGTCATCTCGAGCCACTGGGAGTCAGGTGTTAC  AGGGCCCAGTTAGTACGCCCTCGAGACTCCTGGACTGGATGCACTA | |
| **Mutagenesis primers** | | |
| Name of primer | Sequence (5’-3’) | |
| c.3538-1G>A-F | GCAAGCGGAACCACCACAGACTGAC | |
| c.3538-1G>A -R | ATGGATAGAGACACAAAGATAAGTG | |
| c.839-1G>A-F | ACAACAAGTGCAGAGGACTTTTTCTAT | |
| c.839-1G>A-R | GGGAGGCGAGGAAATAAGGCATGAG | |
| c.4852+11A>G-F | TGGCGGCAGGAGAGCTACATCACTC | |
| c.4852+11A>G-R | GCTTACCTATCATCGGCTGTAACAC | |
| c.2090+3A>T-F | AGGTTACTAACGTGCCCGCCAGCTGC | |
| c.2090+3A>T-R | GGATGGGTAGCCAGCTGCACTGATTC | |
| c.1322+5G>A-F | TAGACCTGCCGCCTGTCTCGCTCTATC | |
| c.1322+5G>A-R | CCCATAGATGTCCAGGACCCCGATG | |
| c.2414+5G>T-F | TGATCCAGACTGTGGCAGCTCAGGGC | |
| c.2414+5G>T-R | CCTGCGGGCCAGGTGTCCCCGGCAG | |
| c.2349A>G-F | TGAAGTATCACAGGCTGAAGGGGGCTAC | |
| c.2349A>G-R | CCTTCTGCAGCCATCCCCGGACAGT | |
| c.1669-35A>C-F | ACTACCGACTGTGTCTCTCTCTGGTTGC | |
| c.1669-35A>C-R | GCTTCAGCAGTTTCTCAACCACTTTC | |
| c.455+8T>C-F | GAACGTCCTCAGCCCAGAAGTCCTC | |
| c.455+8T>C-R | TCACCTGGCCATCTGCTTGTAGGC | |
| c.2415-6C>G-F | GCTGCCCAGGCTGGCTGAGCACCTGC | |
| c.2415-6C>G-R | AAGAGGAAACAGGCAGTGTCAGGGAG | |
| c.3045+3A>T-F | GTTTGCTCAGGCCAGAGGGGCAGGCGCTG | |
| c.3045+3A>T-R  c.613-11G>A-F  c.613-11G>A-R  c.4221G>A-F  c.4221G>A-R | CTTCCTCAGCTCATCTTTCTCCCTGCTGTGGG  TCTAGTCTGTGCAGGCCATTGGAAAT  CAGACAAAGGTATGAAAAAAAAAGAGGGC  TCTAGTGGGTAGCCATGTCTAGTGGA  TTCTCGTTGGTCAGCCGGGATATTTC | |
| **Primers used for amplification of minigene transcripts** | | |
| Name of primer | Sequence (5’-3’) | |
| T7 | TAATACGACTCACTATAGGG | |
| BGHR | TAGAAGGCACAGTCGAGG | |
| **qPCR primers** | | |
| Name of primer | Sequence (5’-3’) | |
| 1322-qPCR-F | ACCCTACCAGCCCCAGGATG | |
| 1322-qPCR-R | CAACCGCGACCCATAGATGT | |
| 2349-qPCR-F | ACCCTACCAGGACCCCGACA | |
| 2349-qPCR-R | CAACCGCGACCTGCGGGCC | |
| 1669-qPCR-F | ACCCTACCAGGTGGAGTACC | |
| 1669-qPCR-R  3045-qPCR-F  3045-qPCR-R  4221-qPCR-F  4221-qPCR-R | CAACCGCGACCTTGCTGGCC  ACCCTACCAGAACAAAGAGT  GCAGCTCAACCGCGACCTTCC  ACCCTACCAGGCTGCTGGAG  CAACCGCGACCAGATTCTCG | |
| **Primers used for amplification of *MYO5B* transcripts in blood** | | |
| Name of primer | | Sequence (5’-3’) |
| E3/4-F  E8/9-R | | CACTTACTGTGGTATCGTACTTG  TCACTCCGAGGAGTGTGAAG |
