# Supplementary material for: Splicing Analysis of MYO5B Noncanonical Variants in Patients with Low Gamma-Glutamyltransferase Cholestasis
Source: Hum Mutat. 2023 Jul 27;2023:8848362. doi: 10.1155/2023/8848362 (PMC11918961; doi:10.1155/2023/8848362)
Supplement: Supplementary 7 — Table S2: performance assessment of in silico prediction tools on experimentally validated variants. [file 8848362.f7.docx]

**Table S2 Performance assessment of *in silico* prediction tools on experimentally validated variants (n = 11).**

| *In silico* prediction tools | Accuracy | Positive predictive value | Negative predictive value |
| --- | --- | --- | --- |
| Human Splicing Finder* | 0.9091 | 0.8889 | 1.0000 |
| Splice AI (>0.2) | 0.9091 | 1.0000 | 0.7500 |
| Varseak (>2) | 0.8182 | 1.0000 | 0.6000 |
| MutationTaster* | 0.4545 | 0.6667 | 0.2000 |
| MaxEntScan (≥ 15%) | 0.7273 | 0.8571 | 0.5000 |

^*^All interpretations, except for No significant impact on splicing signals or No abrogation of potential splice sites, were deemed to predict altered splicing.

Accuracy = (true positives + true negatives) / (all variants)

Positive predictive value = true positives / (true positives + false positives)

Negative predictive value = true negatives / (true negatives + false negatives)
