# Supplementary material for: Splicing Analysis of MYO5B Noncanonical Variants in Patients with Low Gamma-Glutamyltransferase Cholestasis
Source: Hum Mutat. 2023 Jul 27;2023:8848362. doi: 10.1155/2023/8848362 (PMC11918961; doi:10.1155/2023/8848362)
Supplement: Supplementary 3 — Figure S3: the Sanger sequencing data of transcripts identified in minigenes. [file 8848362.f3.pdf]

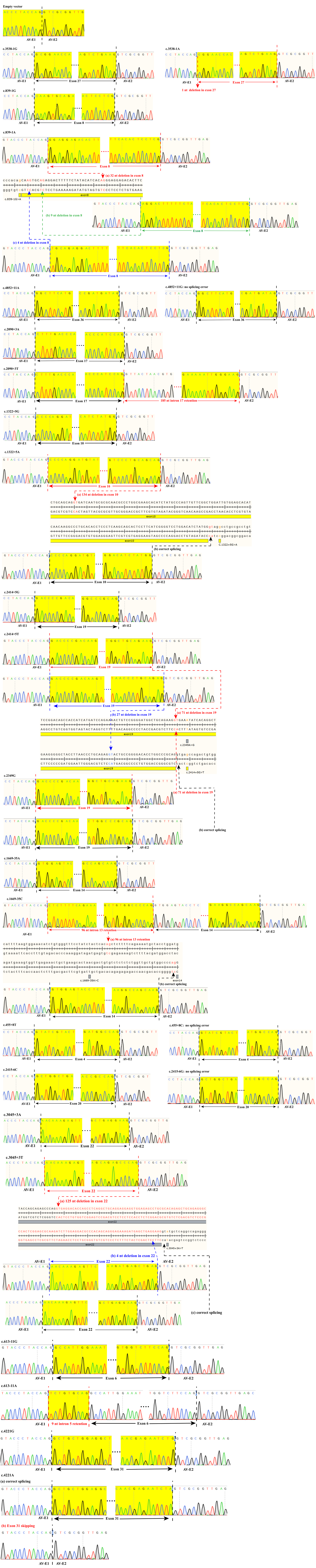

Figure S3. The Sanger sequencing data of transcripts identified in minigenes.
